# Association between night shift work and methylation of a subset of immune-related genes

**DOI:** 10.3389/fpubh.2022.1083826

**Published:** 2023-01-12

**Authors:** Luca Ferrari, Paola Monti, Chiara Favero, Michele Carugno, Letizia Tarantini, Cristina Maggioni, Matteo Bonzini, Angela Cecilia Pesatori, Valentina Bollati

**Affiliations:** ^1^EPIGET Lab, Department of Clinical Sciences and Community Health, Università Degli Studi di Milano, Milan, Italy; ^2^Occupational Health Unit, Fondazione IRCCS Ca' Granda Ospedale Maggiore Policlinico, Milan, Italy

**Keywords:** night shift work, DNA methylation, inflammation, HERV, immune-related genes

## Abstract

**Introduction:**

Night shift (NS) work has been associated with an increased risk of different conditions characterized by altered inflammatory and immune responses, such as cardio-metabolic and infectious diseases, cancer, and obesity. Epigenetic modifications, such as DNA methylation, might mirror alterations in biological processes that are influenced by NS work.

**Methods:**

The present study was conducted on 94 healthy female workers with different working schedules and aimed at identifying whether NS was associated with plasmatic concentrations of the inflammatory proteins NLRP3 and TNF-alpha, as well as with DNA methylation levels of ten human endogenous retroviral (HERV) sequences, and nine genes selected for their role in immune and inflammatory processes. We also explored the possible role of the body mass index (BMI) as an additional susceptibility factor that might influence the effects of NS work on the tested epigenetic modifications.

**Results and discussion:**

We observed a positive association between NS and NLRP3 levels (*p*-value 0.0379). Moreover, NS workers retained different methylation levels for *ERVFRD-1* (*p*-value = 0.0274), *HERV-L* (*p*-value = 0.0377), and *HERV-P* (*p*-value = 0.0140) elements, and for *BIRC2* (*p*-value = 0.0460), *FLRT3* (*p*-value = 0.0422), *MIG6* (*p*-value = 0.0085), and *SIRT1* (*p*-value = 0.0497) genes. We also observed that the BMI modified the relationship between NS and the methylation of *ERVE, HERV-L*, and *ERVW-1* elements. Overall, our results suggest that HERV methylation could pose as a promising biomolecular sensor to monitor not only the effect of NS work but also the cumulative effect of multiple stressors.

## 1. Introduction

In modern “24-h society,” flexible working schedules are becoming increasingly common, with the healthcare industry having one of the highest percentages of night shift (NS) workers to guarantee continuous assistance to patients (1, 2). However, irregular sleep/wake patterns and chronic sleep deprivation could result in disruption of circadian rhythms, i.e., biochemical and behavioral fluctuations synchronized with external 24 h light/dark cycles, with detrimental consequences on both physical and mental health; indeed, circadian rhythms control a wide array of cellular processes, including metabolic homeostasis and innate immunity (3). Therefore, it is not surprising that NS work has been associated with an increased risk of several pathological conditions characterized by altered immune and inflammatory responses, such as infectious diseases, cancer, obesity, and cardiometabolic diseases (4, 5). In this scenario, the identification of molecular modifications mirroring such a complex pattern of biological processes affected by NS work could be of great interest. Besides, this could also help monitor the efficacy of health promotion strategies aimed at reducing NS detrimental effects. Epigenetic modifications such as DNA methylation (i.e., the covalent addition of a methyl group to the C5 position of cytosines, generally occurring at CpG sites) may be suitable candidates since they are known to modulate gene expression in response to a wide array of environmental and lifestyle stressors (6–8). A growing body of literature has reported that NS workers retain an altered methylation profile of clock genes, i.e., the set of genes controlling circadian rhythmicity (9–12). Also, genes involved in the inflammatory process (13, 14) and cancer development (3, 14, 15) are differentially methylated in subjects working at night. Such methylation modifications could also be exacerbated by additional stressful conditions associated with systemic low-grade inflammation, such as being overweight (16).

In this framework, human endogenous retroviruses (HERVs) are one of the most abundant classes of repetitive elements (REs), accounting for 8% of the human genome (17). These sequences originated from ancestral retroviruses that infected the primates' germ line and integrated into the host genome, thus being vertically transmitted across generations; however, the progressive accumulation of sequence changes within HERVs has re-shaped them into novel elements at the service of the host cell innate immune system, modulating the inflammatory response after different environmental triggers (18, 19).

Based on this evidence, HERV sequences may likely respond to NS work by changing their methylation status, with potential implications in the innate immune/inflammatory processes. Being NS work a potential risk factor for infectious and cancer diseases, the immune escape mechanisms might also be involved. Indeed, environmental stressors can affect the capacity of pathogen and tumor cells to evade the immunogenic response by altering the host defense mechanisms (20, 21).

To test this hypothesis, we designed a cross-sectional study to investigate the effects of NS work on inflammation and the methylation of HERV elements and a subset of immune escape-related genes. The analyses were conducted on blood samples of healthy nurses with different NS working schedules, who have been recruited in the context of previous research (15, 22). First, we evaluated whether NS work is associated with the plasmatic concentration of the NLR family pyrin domain containing 3 (NLRP3) and the tumor necrosis factor-alpha (TNF-alpha) inflammatory markers. Second, we tested the association between NS work and the methylation of a panel of 10 HERV elements and 10 genes involved in immune evasion. Finally, we explored the possible role of the body mass index (BMI) as an additional susceptibility factor, potentially influencing the association of NS work with gene methylation.

## 2. Materials and methods

### 2.1. Study population and blood sample collection

The study population consisted of 97 female nurses working at the Fondazione IRCCS Ca' Granda Policlinico Hospital in Milan, Italy, who were enrolled voluntarily in the context of previous research works, as extensively described in previous studies (15, 22). The study was conducted according to the guidelines of the Declaration of Helsinki and approved by the Institutional Review Board Comitato Etico Milano Area 2 of the Fondazione IRCCS Ca' Granda Ospedale Maggiore Policlinico (approval number 702_2015). As previously reported (15), eligibility criteria included: being a female of Caucasian ethnicity, age 30–45 years, and with a length of service of at least 1 year. Subjects were excluded if, at the time of the enrolment were affected by cancer, neurological diseases (e.g., multiple sclerosis, Alzheimer's or Parkinson's disease, epilepsy), or acute relapses of systemic diseases (e.g., cardiovascular diseases, diabetes). Other exclusion criteria were antihypertensive or steroid drug assumption, pregnancy or menopausal status, and body mass index (BMI) >30. These criteria were adopted to avoid any confounding due to reverse causation (23).

After giving signed informed consent, all participants were administered a semi-structured interview to collect personal data about demographics, lifestyle, and medical conditions/treatments, as well as information about their shift work schedules. Current night shift (NS) workers (*N* = 46), i.e., nurses working NS for at least 2 years, were matched by age and length of service to day shifts (DS) workers. NS workers include former NS workers, i.e., nurses who have worked NS for at least 2 years and who have quit it for <1 year up to 20 years before recruitment, as well as nurses who have never worked NS.

Each subject agreed to donate a 12 ml blood sample, which was drawn in the morning (9.00–10.30 a.m.) at the end of the night shift (for NS nurses) or the beginning of the working day (for day shifters). Body parameters collected in the questionnaire were used to calculate the body mass index (BMI), i.e., weight divided by square height (kg/m^2^), taken as an indicator of metabolic health. Complete outcome data (see below) could not be assessed for three subjects, and we thus performed our analyses on 94 nurses.

### 2.2. Blood processing and quantification of plasma NLRP3 and TNF-alpha

Blood samples were collected in EDTA tubes and centrifuged at 1,200 g for 15 min to separate plasma, buffy coat, and red blood cell fractions within 4 h after withdrawal. Plasma aliquots were stored at −80°C until use, making sure to avoid repeated freeze-thaw cycles.

The quantification of plasma NLRP3 was carried out using a commercial enzyme-linked immunosorbent assay (ELISA) kit (cat. MBS3802246, MyBioSource), according to the manufacturer's instructions. The assay was performed using 10 μl of plasma for each subject. The quantification of plasma TNF-alpha was carried out using the Human TNF-alpha Quantikine HS ELISA kit (cat. HSTA00E, R&D Systems) according to the manufacturer's instruction, using 50 μl plasma samples for each subject. A Synergy HT-BioTek spectrophotometer was used to read the optical density at 450 nm. A blank correction was applied for both assays. For TNF-alpha measurement, we applied an additional wavelength correction at 540 nm, as suggested in the assay protocol. Protein concentration (pg/ml) was determined from the standard curve (*R*^2^ > 0.99). As each sample and standard were tested in duplicate, the mean value of the two runs was used in the statistical analysis.

### 2.3. DNA methylation analysis

DNA extraction from Buffy coat and sodium bisulfite treatment, followed by amplification of the DNA sequences of interest and pyrosequencing, were performed according to the procedure described in Monti et al. (16). PCR cycling condition and primer sequences used for PCR amplification and pyrosequencing are reported in Supplementary Table S1. CpG sites were queried within regulatory regions (promoter and enhancer regions) of the following genes: *ERVE, ERVFRD-1, ERVH, ERVK, HERV-L, HERV-P, ERV3-1, ERV9-1, ERVW-1, HRES1, BIRC2, FLRT3, GAL9, IDO1, LPHN1, MIG6, NLRC5, SIRT1*, and *XIAP*. Every sample was measured twice for each gene to test the reproducibility of the experimental setting.

For each gene, methylation levels were calculated as the percentage of methylated cytosines out of the total number of cytosines (5-methyl-cytosine + unmethylated cytosines) at each CpG site of interest. The designed assays allow the assessment of a variable number of CpG sites (1–5). Coefficients of variation for each assay are as follows: *ERVE* = 0.02, *ERVFRD-1* = 0.01, *ERVH* = 0.01, *ERVK* = 0.03, *ERVL* < 0.01, *HERV-P* = 0.02, *ERV3-1* = 0.01, *ERV9-1* = 0.03, *ERVW-1* = 0.01, *HRES1* = 0.07, *BIRC2* = 0.18, *FLRT3* = 0.09, *GAL9* = 0.12, *IDO1* = 0.03, *LPHN1* = 0.06, *MIG6* = 0.03, *NLRC5* = 0.04, *SIRT1* = 0.14, and *XIAP* = 0.01.

### 2.4. Statistical analysis

Collected data were summarized by standard descriptive statistics, and a graphical inspection of the main variables of interest was performed to examine their distribution. Continuous variables were expressed as mean ± standard deviation (SD), whereas categorical data were reported as frequencies (%). The study population was divided into different groups: current NS workers vs. colleagues who are currently not working DS (“Yes” vs. “No”), current + former NS workers (“ever”) vs. colleagues who have never worked NS (“never”), and “current” vs. “former” vs. “never” shifters.

Baseline characteristics by current NS work (yes vs. no) were reported and compared with a *t*-test for continuous variables and the chi-square test or Fisher's exact test as appropriate for categorical variables.

To test the association of NS work with the concentration of plasmatic NLRP3 and TNF-alpha, we applied ANCOVA models adjusted for age, BMI, smoking habits, use of oral contraceptives, general health medications, and ELISA plate.

To estimate the effect of NS work on DNA methylation for the genes of interest, we fitted linear mixed-effect models to consider intra-individual correlation due to repeated-measure data structure. Models were adjusted for age, BMI, smoking habits, years of NS work, as well as run, CpG position, and their interaction (run^*^position). DNA methylation measurements for each subject were run in duplicate. The pyrosequencing-based DNA methylation analysis tested a variable number of CpG positions according to CpG density in the promoter assay. Linear mixed-effect models were used to account for each CpG dinucleotide position (as a random effect) measured in the two runs. An unstructured covariance structure was used to model within-subject errors. Methylation mean levels were calculated as marginal means.

To examine the potential modifying effect of BMI on the association between NS and gene methylation, we added an interaction term between BMI and NS work in each multivariable linear mixed-effect model. Differences between study groups were indicated as β mean differences, standard error (SE), and 95% confidence intervals (CI). We evaluated whether the effect of NS work on methylation levels differs depending on BMI levels. The cut-offs selected for BMI were: 25th percentile (19.8 kg/m^2^), 50th (22.0 kg/m^2^), 75th percentile (24.2 kg/m^2^), and 95th percentile (29.1 kg/m^2^).

Statistical analyses were performed with SAS software (version 9.4; SAS, Cary, NC, USA). A two-sided *p*-value of 0.05 was considered statistically significant.

## 3. Results

### 3.1. Characteristics of the study population

Out of 94 eligible subjects, 44 were working NS at the enrollment (i.e., current NS), and were matched by age and length of service with 50 female colleagues who worked day shifts (DS; Table 1). Among the latter, 28 had never worked NS, and 22 were former NS workers. The majority of study participants had a healthy BMI [i.e., 18.5 ≤ BMI ≤ 24.9 kg/m^2^ (24)]. No significant differences were observed between DS and NS workers regarding age, length of service, BMI, smoking habits, and pharmacological treatments (oral contraceptive use, general health medications, anti-depressive medications, and sleeping pill). The only exception was the family situation, regarding the marital status (*p*-value < 0.0001) and the number of children (*p*-value = 0.0026).

**Table 1:** Characteristics of the study participants.

**Characteristics**	**Total**	**Current night shift**		***p*-value**

		**Yes (*****N*** = **44)**	**No (*****N*** = **50)**	
Age, years	35.8 ± 5.4	35.1 ± 5.4	36.5 ± 5.4	0.2166
Lenght of service, years	11.6 ± 6.8	10.4 ± 5.9	12.7 ± 7.3	0.0917
Lenght of service with night shift work, years	5.5 [0; 9]	8 [5; 12]	0 [0; 7]	<0.0001
BMI*, kg/m^2^*	22.5 ± 3.3	23.0 ± 3.2	22.2 ± 3.3	0.2529
**Smoking status**				
Never/former	66 (69.5%)	29 (64.5%)	37 (74.0%)	0.5597
Current smoker	26 (27.4%)	14 (31.1%)	12 (24.0%)	
Missing	3 (3.2%)	2 (4.4%)	1 (2.0%)	
**Marital status**				
Not married	13 (13.7%)	-	13 (26.0%)	<0.0001
Married	58 (61.0%)	28 (63.6%)	30 (60.0%)	
Divorced/widow	21 (22.3%)	16 (36.4%)	5 (10.0%)	
Missing	1 (1.0%)	-	2 (4.0%)	
**Number of children**				
0	67 (71.3%)	39 (88.7%)	28 (56.0%)	0.0026
1	12 (12.8%)	2 (4.5%)	10 (20.0%)	
2	11 (11.7%)	3 (6.8%)	8 (16.0%)	
3	4 (4.2%)	-	4 (8.0%)	
**Oral contraceptive use**				
Yes	34 (35.8%)	18 (40.0%)	16 (32.0%)	0.7687
No	57 (60.0%)	25 (55.6%)	32 (64.0%)	
Missing	4 (4.2%)	2 (4.4%)	2 (4.0%)	
**General health medications**				
Yes	36 (38.3%)	21 (47.7%)	15 (30.0%)	0.0777
No	58 (61.7%)	23 (52.3%)	35 (70.0%)	
**Antidepressive medications**				
Yes	4 (4.3%)	3 (6.8%)	1 (2.0%)	0.3372
No	90 (95.7%)	41 (93.2%)	49 (98.0%)	
**Sleeping pill**				
Yes	4 (4.3%)	2 (4.5%)	2 (4.0%)	1
No	90 (95.7%)	42 (95.5%)	48 (96.0%)	

Continuous variables are expressed as mean ± standard deviation (SD) and discrete variables are expressed as counts (%). We applied Student's *t*-test for normal continuous variables and Chi-square or Fisher's exact tests for categorical variables.

### 3.2. Association between night shift work and plasma levels of NLRP3 and TNF-alpha

The average NLRP3 concentration calculated on the whole study population was 15.38 ± 2.44 pg/ml and the TNF-alpha concentration was 0.8 ± 0.4 pg/ml. Then, we tested the possible association between NS work and the plasmatic mean concentration of NLRP3 and TNF-alpha (Table 2). We observed a positive association with NLRP3 levels (*p*-value = 0.0379). We further tested whether having ever been a shift worker (i.e., ever vs. never) and being currently a NS worker (i.e., current vs. former vs. never) was associated with NLRP3 and TNF alpha concentrations (Supplementary Table S2).

**Table 2:** Association between NS work and NLRP3 and TNF alpha.

**Protein**	**Current night shift work**	**Mean**	**95% CI**		***p*-value**
NLRP3	Yes	16.24	15.53	16.95	0.0379
	No	15.32	14.65	15.99	
TNF-alpha	Yes	0.76	0.67	0.86	0.6844
	No	0.78	0.69	0.89	

Estimates were calculated by a multivariable regression model adjusted for age, BMI, smoking habits, oral contraceptive use, general health medications, and ELISA plate.

### 3.3. Association between current NS work and DNA methylation

We tested whether DNA methylation levels of HERV elements (*ERVE, ERVFRD-1, ERVH, ERVK, ERVL, HERV-P, ERV3-1, ERV9-1, ERVW-1*, and *HRES1*) and immune-related genes *(BIRC2, FLRT3, GAL9, IDO1, LPHN1, MIG6, NLRC5, SIRT1*, and *XIAP*) were different in night and day shifters (Table 3). Statistically significant differences were observed for *ERVFRD-1, ERVL, HERV-P, BIRC2, FLRT3, MIG6*, and *SIRT1*. In particular, we observed a lower percentage of methylated CpGs for *ERVFRD-1, FLRT3*, and *MIG6* in night- compared to day-shifters, whereas *ERVL, HERV-P, BIRC2*, and *SIRT1* methylation was higher in NS nurses.

**Table 3:** Association between current NS work and gene-specific methylation.

**Gene**	**Current NS work**	**Mean methylation (%5 mCpG)**	**SE**	**95% CI**		***p*-value**
*ERVE*	Yes	84.40	0.26	83.88	84.91	0.1092
	No	84.98	0.23	84.52	85.44	
*ERVFRD-1*	Yes	76.91	0.61	75.72	78.11	0.0274
	No	78.82	0.56	77.70	79.93	
*ERVH*	Yes	86.21	0.32	85.57	86.85	0.3709
	No	86.63	0.31	86.02	87.25	
*ERVK*	Yes	57.96	0.50	56.97	58.96	0.8785
	No	57.86	0.46	56.94	58.77	
*ERVL*	Yes	92.32	0.10	92.12	92.53	0.0377
	No	92.01	0.10	91.82	92.21	
*HERV-P*	Yes	64.60	0.87	62.86	66.34	0.0140
	No	61.46	0.82	59.82	63.11	
*ERV3-1*	Yes	98.11	0.24	97.62	98.59	0.5768
	No	98.30	0.22	97.86	98.74	
*ERV9-1*	Yes	79.63	0.63	78.37	80.89	0.4526
	No	78.94	0.61	77.73	80.16	
*ERVW-1*	Yes	94.16	0.15	93.86	94.46	0.8502
	No	94.21	0.14	93.93	94.49	
*HRES1*	Yes	6.41	0.10	6.20	6.61	0.1473
	No	6.20	0.09	6.02	6.38	
*BIRC2*	Yes	4.36	0.08	4.19	4.52	0.0460
	No	4.12	0.08	3.96	4.27	
*FLRT3*	Yes	1.55	0.08	1.38	1.71	0.0422
	No	1.79	0.08	1.64	1.95	
*GAL9*	Yes	2.42	0.07	2.28	2.55	0.7748
	No	2.39	0.07	2.26	2.52	
*IDO1*	Yes	5.28	0.22	4.84	5.71	0.8198
	No	5.35	0.21	4.93	5.76	
*LPHN1*	Yes	1.16	0.03	1.09	1.23	0.6221
	No	1.14	0.03	1.07	1.20	
*MIG6*	Yes	3.82	0.12	3.59	4.05	0.0085
	No	4.26	0.11	4.04	4.48	
*NLRC5*	Yes	38.20	1.08	36.05	40.34	0.6937
	No	38.80	1.00	36.83	40.76	
*SIRT1*	Yes	8.64	0.47	7.72	9.56	0.0497
	No	7.32	0.41	6.51	8.13	
*XIAP*	Yes	29.62	0.44	28.76	30.48	0.4490
	No	30.09	0.42	29.27	30.92	

Methylation means were calculated as marginal means from linear mixed-effect regression model adjusted for age, BMI, smoking habits, years of NS work, run, position, and their interaction (run^*^position).

### 3.4. Effect of BMI on the association between current NS work and DNA methylation

**Figure 1:**
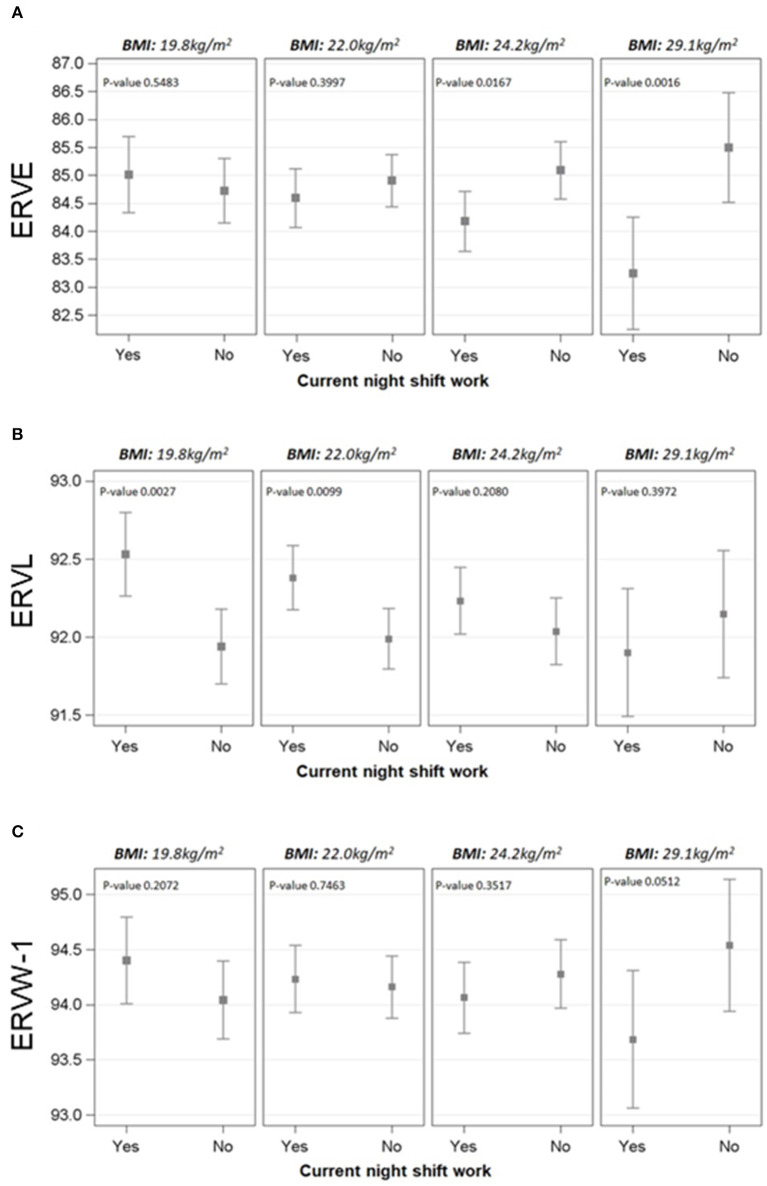
Effect modification of BMI on the association between NS work and methylation of ERVE **(A)**, ERVL **(B)**, and ERVW-1 **(C)**. The cut-offs selected for BMI were 25th, 50th, 75th, and 95th percentile (19.8, 22.0, 24.2, and 29.1 kg/m^2^, respectively). Linear mixed regression models were adjusted for age, BMI, smoking habits, length of service with NS work, the interaction term BMI*current NS work, run, position, and their interaction. Methylation levels were calculated as marginal means.

As overweight could represent an additional susceptibility factor for NS workers, we evaluated the modification effect of BMI on the association between current NS work and DNA methylation of the genes. Interestingly, the only significant interactions between current NS work and BMI were observed for HERV genes, in particular for *ERVE, ERVL*, and *ERVW-1* (*p*-values = 0.007, =0.029, and =0.032, respectively) (Figure 1). The strength of these associations was evaluated at four fixed levels of BMI (25th percentile: 19.8 kg/m^2^, 50th percentile: 22.0 kg/m^2^, 75th percentile: 24.2 kg/m^2^, and 95th percentile: 29.1 kg/m^2^).

For *ERVE*, the percentage of mCpG sites estimated at BMI = 24.2 kg/m^2^ was found to be lower in NS workers if compared to DS (β = −0.91; *p*-value = 0.0167). The difference becomes even wider at BMI = 29.1 kg/m^2^ (β = −2.25; *p-*value = 0.002) and it was not significant for lower levels of BMI_._ On the contrary, the association between NS and *ERVL* methylation was different for lower-mid BMI values, with higher methylation levels in NS if compared to DS at BMI = 19.8 kg/m^2^ (β = 0.59; *p*-value =0.003) and BMI = 22.0 kg/m^2^ (β = 0.39; *p*-value = 0.010). Regarding *ERVW-1*, a slight difference was observed at BMI = 29.1 kg/m^2^, with NS workers having lower methylation levels (β = −0.85; *p*-value = 0.052). The associations between current NS work and methylation levels at selected BMI values are reported in Supplementary Table S3.

## 4. Discussion

In the present study conducted on 94 female workers with different working schedules, we first evaluated the association of NS work with plasmatic concentrations of NLRP3 and TNF-alpha.

NLRP3 and TNF-alpha were chosen as paradigmatic indicators of innate immune and inflammatory alterations (25). We found that plasmatic levels of NLRP3 were increased in NS nurses compared to colleagues with daily schedules. NLRP3 is a crucial player in host immune defenses by mediating the activation of caspase-1 and the secretion of proinflammatory cytokines IL-1β and IL-18 (26). NLRP3 expression is controlled by many endogenous and exogenous factors, including circadian rhythms (27). A recent study reported that the core clock component NR1D1 negatively regulates NLRP3 expression and activation, as well as the downstream release of IL-1β and IL-18 (28). Of note, circadian rhythm disruption results in impaired NLRP3 levels, as supported by a study reporting NLRP3 overexpression in mice subjected to sleep deprivation (29). On the contrary, we found no difference in plasma TNF-alpha concentrations between the two study groups. TNF-alpha alterations could be expected among NS workers, as the secretion of this proinflammatory cytokine is under circadian control (30). However, data available in the literature are inconsistent, with studies reporting either positive (31) or negative (30) associations between NS and plasmatic TNF-alpha levels, while others observed non-significant differences (32–34). The recent finding that TNF-alpha knockout and wild-type mice do not respond differently to sleep deprivation (35) led to speculation that other pathways (such as the NLRP3 one) might have a greater impact on the response to circadian disruption (25, 35).

Being DNA methylation affected by environmental triggers, including NS (22, 36), we also examined the methylation levels of HERV elements, which are implied in immune and inflammatory responses (18).

Three HERV genes were differentially methylated between NS and DS workers. In particular, *ERVFRD-1* methylation was lower, while *ERVL* and *HERV-P* methylation levels were higher in nurses working at night. HERVs derive from ancestral retroviruses that infected the primates' germ line and subsequently integrated into the human genome, thus becoming endogenous elements stably inherited across generations (18). Indeed, the structural organization of HERVs resembles that of retroviral DNA, with the *gag, pol*, and *env* genes sided by two long terminal repeats (LTRs) with regulatory functions. However, the progressive accumulation of sequence changes within ancestral retroviral elements has led to the “domestication” of HERVs, leading in most cases to the loss of protein-coding capacity and the acquisition of novel functions at the interface between the “self” and “non-self.” HERVs are emerging as important players in the host's innate immune system by modulating the inflammatory response to pathogens and other external triggers (18). Being non-unique sequences, HERVs are generally subjected to transcriptional silencing to prevent genomic instability (37). Nevertheless, changes in HERV methylation levels have been reported in response to many proinflammatory stressors (e.g., environmental toxicants and lifestyle stressors) with possible pathological outcomes (19). Given their numerous and largely unexplored physiological functions, their alterations have been implicated in cancer, as well as in several autoimmune and inflammatory diseases (38, 39). HERVs can act as pathogen-associated molecular patterns, triggering both innate and adaptive immune responses (18, 40–42) by providing antigenic epitopes recognized by lymphocytes and stimulating the onset of specific T- and B- cells (43, 44). These mechanisms might be crucial for the involvement of HERVs in autoimmune diseases, such as multiple sclerosis (*ERVW-1*), systemic lupus erythematosus (*ERVE* and *HRES*), rheumatoid arthritis (*ERVK*), and amyotrophic lateral sclerosis (*ERVK*) (45, 46).

On the other hand, HERVs have also been involved in immune response downregulation, starting from their crucial role in maternal immune tolerance to their suggested protective action against undue immune activation (41). The immune response downregulation underlies HERV involvement in tumorigenesis. Interestingly, aberrant methylation and expression of HERV elements have been reported in many cancer types, including breast, colorectal, and prostate cancer (39, 46), for which an increased risk has been reported also in association with NS work (47, 48).

We observed that *ERVE, ERVW-1*, and *ERVL* were differentially methylated depending on the BMI. In particular, *ERVE* and *ERVW-1* were differentially methylated between NS and DS at high BMI values, while *ERVL* methylation was different for lower-mid BMI values. These results suggest that the overweight condition might be an additional susceptibility factor interacting with NS in fostering an altered immune/inflammatory response modulation. We recently reported an increase in age acceleration per year in NS work in subjects with overweight/obesity (22). Thus, HERV sequences might be considered sensitive indicators of immune alterations caused by multiple environmental stress factors.

We further investigated the association between NS work and the methylation of a subset of immune-related genes. We found that *BIRC2* and *SIRT1* methylation was higher in NS nurses. *BIRC2* (Baculoviral IAP Repeat Containing 2) encodes a member of the inhibitors of apoptosis (IAP) family of proteins. *BIRC2* expression is downregulated by promoter hypermethylation (49, 50), which was also associated with increased IL-1β production (49, 51). Similarly, hypermethylation of the *SIRT1* gene (encoding the Sirtuin 1, a deacetylase whose downregulation has been found in many disease conditions) correlates with lower transcript levels and with an increased risk of inflammatory and metabolic diseases (52, 53). SIRT1 affects multiple biological processes by de-acetylating a variety of proteins including histones and non-histone proteins. Alterations to SIRT1 methylation, expression, and activity were linked to inflammatory diseases (52). It is therefore possible that NS-induced hypermethylation of such genes might determine an increased inflammation and susceptibility to related pathological conditions.

On the contrary, *FLRT3* and *MIG6* methylation levels were reduced in NS workers. FLRT3 (Fibronectin leucine-rich transmembrane protein 3) is a protein involved in cell adhesion and receptor signaling. Although to our knowledge methylation levels of the *FLRT3* gene have never been investigated before, *FLRT3* overexpression was found to be protective toward tumorigenesis as it promotes apoptosis and suppresses epithelial-mesenchymal transition, as well as colorectal cancer cell proliferation, migration, and invasion (54). Besides, *FLRT3* is overexpressed in breast cancer cells and participates in immune escape processes by regulating the immune receptor Tim-3 pathway (55). Also, *MIG6* (Mitogen-inducible gene-6, also known as *ERRFI1*) has been principally studied in the context of cancer biology, where it poses as a tumor suppressor that inhibits EGFR signaling. *MIG6* hypermethylation results in transcriptional downregulation (56), which has been observed in many cancer types; however, the impact of DNA methylation on *MIG6* expression could depend on cancer type, as treatment with DNA methyltransferase inhibitor 5-aza-2′-deoxycytidine did not always affect *MIG6* expression (57).

In the present study, we selectively included workers without major pathological conditions at the time of enrollment to specifically focus on the effects of NS work on DNA methylation and exclude possible confounding due to ongoing disease states. The modifications observed might mirror alterations in biological processes affected by NS work. Future functional studies are needed to evaluate the effects of DNA methylation differences reported in the present associative study. Our findings suggest that DNA methylation of HERV elements and immune-related genes could be a promising marker to highlight the complex pattern of inflammatory alterations found in NS workers (supported by plasmatic NLRP3 increase). However, prospective studies are needed to explore the causal relationship between NS exposure and the observed biological modifications, and eventually evaluate their potential as biomarkers of NS-related diseases.

We acknowledge some limitations of the present study. First, the study population is quite small, especially when it comes to considering BMI classes (the number of study participants with BMI>29.1 kg/m^2^ is very small). Second, we selected the genes of interest based on current evidence in the literature, and not based on unbiased genome-wide methylation analysis; therefore, we could have missed additional methylation alterations occurring in uninvestigated regions. Third, the observed methylation differences between night and day shifters are slight. These results are expected upon exposure of a healthy population to environmental/occupational factors and have emerged thanks to the high precision technology employed and the analytical method applied. However, it is possible that the reduced variations detected might have been underestimated due to the analysis of bulk samples (i.e., buffy coat cell populations). Future studies should be conducted to clarify this aspect.

## 5. Conclusion

In the present study, although explorative and based on a small sample, we observed that exposure to NS work is associated with altered modulation of immune and inflammatory-related factors. Moreover, our findings indicate DNA and in particular HERV methylation as a potential biomolecular sensor to monitor the cumulative effect of multiple stressors (NS and metabolic alterations).

## Data availability statement

The original contributions presented in the study are included in the article/Supplementary material, further inquiries can be directed to the corresponding author.

## Ethics statement

The study was conducted according to the guidelines of the Declaration of Helsinki and approved by the Institutional Review Board Comitato Etico Milano Area 2 of the Fondazione IRCCS Ca' Granda Ospedale Maggiore Policlinico (approval number 702_2015). The patients/participants provided their written informed consent to participate in this study.

## Author contributions

LF, ACP, and VB contributed to the conception and design of the study. LF, PM, and LT performed experimental analyses. CF and MC organized the database and performed the statistical analysis. CM and MB enrolled the subjects. LF and PM wrote the first draft of the manuscript. LF and VB supervised the whole study. All authors contributed to the manuscript revision, read, and approved the submitted version.

## References

[1] Costa G. Shift work and health: current problems and preventive actions. Saf Health Work. (2010) 1:112–23. 10.5491/SHAW.2010.1.2.112 22953171 PMC3430894

[2] Wilson JL. The impact of shift patterns on healthcare professionals. J Nurs Manag. (2002) 10:211–9. 10.1046/j.1365-2834.2002.00308.x 12100600

[3] Labrecque N Cermakian N. Circadian clocks in the immune system. J Biol Rhythms [Internet]. (2015) 30:277–90. 10.1177/0748730415577723 25900041

[4] Torquati L Mielke GI Brown WJ Burton NW Kolbe-Alexander TL. Shift work and poor mental health: a meta-analysis of longitudinal studies. Am J Public Health. (2019) 109:E13–20. 10.2105/AJPH.2019.305278 31536404 PMC6775929

[5] Kecklund G Axelsson J. Health consequences of shift work and insufficient sleep. BMJ. (2016) 355:5210. 10.1136/bmj.i5210 27803010

[6] Rider CF Carlsten C. Air pollution and DNA methylation: effects of exposure in humans. Clin Epigenetics. (2019) 11:7132. 10.1186/s13148-019-0713-2 31481107 PMC6724236

[7] Alegría-Torres JA Baccarelli A Bollati V. Epigenetics and lifestyle. Epigenomics. (2011 ) 3:267. 10.2217/epi.11.22 22122337 PMC3752894

[8] Martin EM Fry RC. Environmental influences on the epigenome: exposure- associated dna methylation in human populations. Annu Rev Public Health. (2018) 39:309–33. 10.1146/annurev-publhealth-040617-014629 29328878

[9] Ritonja JA Aronson KJ Flaten L Topouza DG Duan QL Durocher F. Exploring the impact of night shift work on methylation of circadian genes. Epigenetics. (2021) 3:141. 10.1136/OEM-2021-EPI.141 34825628 PMC9542576

[10] Reszka E Wieczorek E Przybek M Jabłońska E Kałuzny P Bukowska-Damska A. Circadian gene methylation in rotating-shift nurses: a cross-sectional study. Chronobiol Int. (2018) 35:111–21. 10.1080/07420528.2017.1388252 29144171

[11] Bukowska-Damska A Reszka E Kaluzny P Wieczorek E Przybek M Zienolddiny S. Sleep quality and methylation status of core circadian rhythm genes among nurses and midwives. Chronobiol Int. (2017) 34:1211–23. 10.1080/07420528.2017.1358176 29106308

[12] Ritonja JA Aronson KJ Leung M Flaten L Topouza DG Duan QL. Investigating the relationship between melatonin patterns and methylation in circadian genes among day shift and night shift workers. Occup Environ Med. (2022) 3:8111 10.1136/oemed-2021-108111 35501127

[13] Bollati V Baccarelli A Sartori S Tarantini L Motta V Rota F. Epigenetic effects of shiftwork on blood dna methylation. Chronobiol Int. (2010) 27:1093–104. 10.3109/07420528.2010.490065 20636218 PMC3647609

[14] Adams CD Jordahl KM Copeland W Mirick DK Song X Sather CL. Nightshift work, chronotype, and genome-wide DNA methylation in blood. Epigenetics. (2017) 12:833–40. 10.1080/15592294.2017.1366407 28837395 PMC5788410

[15] Carugno M Maggioni C Crespi E Bonzini M Cuocina S Dioni L. Night shift work, DNA methylation and telomere length: an investigation on hospital female nurses. Int J Environ Res Public Health. (2019) 16:2292. 10.3390/ijerph16132292 31261650 PMC6651131

[16] Monti P Iodice S Tarantini L Sacchi F Ferrari L Ruscica M. Effects of PM exposure on the methylation of clock genes in a population of subjects with overweight or obesity. Int J Environ Res Public Health. (2021) 18:1–15. 10.3390/ijerph18031122 33513987 PMC7908270

[17] Lander ES Linton LM Birren B Nusbaum C Zody MC Baldwin J. Initial sequencing and analysis of the human genome. Nature. (2001) 409:860–921. 10.1038/35057062 11237011

[18] Grandi N Tramontano E. Human endogenous retroviruses are ancient acquired elements still shaping innate immune responses. Front Immunol. (2018) 9:2039. 10.3389/fimmu.2018.02039 30250470 PMC6139349

[19] Cho K Lee YK Greenhalgh DG. Endogenous retroviruses in systemic response to stress signals. Shock. (2008) 30:105–16. 10.1097/SHK.0b013e31816a363f 18317406

[20] Franchini AM Lawrence BP. Environmental exposures are hidden modifiers of anti-viral immunity. Curr Opin Toxicol. (2018) 10:54–9. 10.1016/j.cotox.2018.01.004 30035244 PMC6051538

[21] Pozniak J Nsengimana J Laye JP O'Shea SJ Diaz JMS Droop AP. Genetic and environmental determinants of immune response to cutaneous melanoma. Cancer Res]. (2019) 79:2684–96. 10.1158/0008-5472.CAN-18-2864 30773503 PMC6544535

[22] Carugno M Maggioni C Ruggiero V Crespi E Monti P Ferrari L. can night shift work affect biological age? Hints from a cross-sectional study on hospital female nurses. Int J Environ Res Public Health. (2021) 18:10639. 10.3390/ijerph182010639 34682384 PMC8535512

[23] Richardson TG Richmond RC North TL Hemani G Davey Smith G Sharp GC. An integrative approach to detect epigenetic mechanisms that putatively mediate the influence of lifestyle exposures on disease susceptibility. Int J Epidemiol. (2019) 48:887–98. 10.1093/ije/dyz119 31257439 PMC6659375

[24] Weir CB Jan A. BMI Classification Ion Percentile and Cut Off Points. Treasure Island, FL: StatPearls Publishing (2022). 31082114

[25] Zielinski MR Gibbons AJ. Neuroinflammation, Sleep, and Circadian Rhythms. Front Cell Infect Microbiol. (2022) 12:3096. 10.3389/fcimb.2022.853096 35392608 PMC8981587

[26] Kelley N Jeltema D Duan Y He Y. The NLRP3 inflammasome: an overview of mechanisms of activation and regulation. Int J Mol Sci MDPI AG. (2019) 59:3328. 10.3390/ijms20133328 31284572 PMC6651423

[27] Pourcet B Duez H. Circadian control of inflammasome pathways: implications for circadian medicine. Front Immunol. (2020) 11:1630. 10.3389/fimmu.2020.01630 32849554 PMC7410924

[28] Pourcet B Zecchin M Ferri L Beauchamp J Sitaula S Billon C. Nuclear receptor subfamily 1 group D member 1 regulates circadian activity of NLRP3 inflammasome to reduce the severity of fulminant hepatitis in mice. Gastroenterology. (2018) 154:1449–64. 10.1053/j.gastro.2017.12.019 29277561 PMC5892845

[29] Zielinski MR Gerashchenko D Karpova SA Konanki V McCarley RW Sutterwala FS. The NLRP3 inflammasome modulates sleep and NREM sleep delta power induced by spontaneous wakefulness, sleep deprivation and lipopolysaccharide. Brain Behav Immun. (2017) 62:137–50. 10.1016/j.bbi.2017.01.012 28109896 PMC5373953

[30] Liu PY Irwin MR Krueger JM Gaddameedhi S Van Dongen HPA. Night shift schedule alters endogenous regulation of circulating cytokines. Neurobiol Sleep Circadian Rhythm. (2021) 10:63. 10.1016/j.nbscr.2021.100063 33748539 PMC7970107

[31] Cakan P Yildiz S. Effects of half- or whole-night shifts on physiological and cognitive parameters in women. Am J Med Sci. (2020) 360:525–36. 10.1016/j.amjms.2019.12.002 31882159

[32] Van Mark A Weiler SW Schröder M Otto A Jauch-Chara K Groneberg DA. The impact of shift work induced chronic circadian disruption on IL-6 and TNF-alpha immune responses. J Occup Med Toxicol. (2010) 5:18. 10.1186/1745-6673-5-18 20602750 PMC2914774

[33] Copertaro A Bracci M Gesuita R Carle F Amati M Baldassari M. Influence of shift-work on selected immune variables in nurses. Ind Health. (2011) 49:597–604. 10.2486/indhealth.MS1210 21804267

[34] Loef B Nanlohy NM Jacobi RHJ van de Ven C Mariman R van der Beek AJ. Immunological effects of shift work in healthcare workers. Sci Rep. (2019) 9:4816. 10.1038/s41598-019-54816-5 31796836 PMC6890754

[35] Szentirmai É Kapás L. Sleep and body temperature in TNFα knockout mice: the effects of sleep deprivation, β3-AR stimulation and exogenous TNFα. Brain Behav Immun. (2019) 81:260. 10.1016/j.bbi.2019.06.022 31220563 PMC6754767

[36] Ferrari L Carugno M Bollati V. Particulate matter exposure shapes DNA methylation through the lifespan. Clin Epigenetics. (2019) 11:726. 10.1186/s13148-019-0726-x 31470889 PMC6717322

[37] Groh S Schotta G. Silencing of endogenous retroviruses by heterochromatin. Cell Mol Life Sci. (2017) 74:2055–65. 10.1007/s00018-017-2454-8 28160052 PMC11107624

[38] Mustelin T Ukadike KC. How retroviruses and retrotransposons in our genome may contribute to autoimmunity in rheumatological conditions. Front Immunol. (2020) 11:3891. 10.3389/fimmu.2020.593891 33281822 PMC7691656

[39] Alcazer V Bonaventura P Depil S. Human endogenous retroviruses (HERVs): shaping the innate immune response in cancers. Cancers. (2020) 12:610. 10.3390/cancers12030610 32155827 PMC7139688

[40] Wolff F Leisch M Greil R Risch A Pleyer L. The double-edged sword of (re)expression of genes by hypomethylating agents: from viral mimicry to exploitation as priming agents for targeted immune checkpoint modulation. Cell Commun Signal. (2017) 15:0168. 10.1186/s12964-017-0168-z 28359286 PMC5374693

[41] Dupressoir A Lavialle C Heidmann T. From ancestral infectious retroviruses to bona fide cellular genes: role of the captured syncytins in placentation. Placenta. (2012) 33:663–71. 10.1016/j.placenta.2012.05.005 22695103

[42] Hurst TP Magiorkinis G. Activation of the innate immune response by endogenous retroviruses. J Gen Virol. (2015) 96(Pt 6):1207–18. 10.1099/vir.0.000017 26068187

[43] Roulois D Loo Yau H Singhania R Wang Y Danesh A Shen SY. DNA-demethylating agents target colorectal cancer cells by inducing viral mimicry by endogenous transcripts. Cell. (2015) 162:961–73. 10.1016/j.cell.2015.07.056 26317465 PMC4843502

[44] Trela M Nelson PN Rylance PB. The role of molecular mimicry and other factors in the association of human endogenous retroviruses and autoimmunity. APMIS. (2016) 124:88–104. 10.1111/apm.12487 26818264

[45] Buzdin AA Prassolov V Garazha A V. Friends-enemies: endogenous retroviruses are major transcriptional regulators of human DNA. Front Chem. (2017) 5:35. 10.3389/fchem.2017.00035 28642863 PMC5462908

[46] Gröger V Cynis H. Human endogenous retroviruses and their putative role in the development of autoimmune disorders such as multiple sclerosis. Front Microbiol. (2018) 9:265. 10.3389/fmicb.2018.00265 29515547 PMC5826199

[47] Lie JAS Kjuus H Zienolddiny S Haugen A Stevens RG Kjærheim K. Night work and breast cancer risk among Norwegian nurses: assessment by different exposure metrics. Am J Epidemiol. (2011) 173:1272–9. 10.1093/aje/kwr014 21454824

[48] Wegrzyn LR Tamimi RM Rosner BA Brown SB Stevens RG Eliassen AH. Rotating night-shift work and the risk of breast cancer in the nurses' health studies. Am J Epidemiol. (2017) 186:532–40. 10.1093/aje/kwx140 28541391 PMC5856106

[49] Tseng CC Liao WT Wong MC Chen CJ Lee SC Yen JH. Cell lineage-specific methylome and genome alterations in gout. Aging. (2021) 13:3843. 10.18632/aging.202353 33493135 PMC7906142

[50] Zhou XH Lin W Ren YM Liu S Fan BY Wei ZJ. Comparison of DNA Methylation in schwann cells before and after peripheral nerve injury in rats. Biomed Res Int. (2017) 2017:3268. 10.1155/2017/5393268 28459064 PMC5385226

[51] Saleem M Qadir MI Perveen N Ahmad B Saleem U Irshad T. Inhibitors of apoptotic proteins: new targets for anticancer therapy. Chem Biol Drug Des. (2013) 82:243–51. 10.1111/cbdd.12176 23790005

[52] Yang Y Liu Y Wang Y Chao Y Zhang J Jia Y. Regulation of SIRT1 and its roles in inflammation. Front Immunol. (2022) 13:872. 10.3389/fimmu.2022.831168 35359990 PMC8962665

[53] Heidari L Ghaderian SMH Bastami M Hosseini S Alipour Parsa S Heidari S. Reverse expression pattern of sirtuin-1 and histone deacetylase-9 in coronary artery disease. Arch Physiol Biochem. (2020) 3:7100. 10.1080/13813455.2020.1797100 32758009

[54] Yang M Li D Jiang Z Li C Ji S Sun J. TGF-β-induced FLRT3 attenuation is essential for cancer-associated fibroblast-mediated epithelial-mesenchymal transition in colorectal cancer. Mol Cancer Res. (2022) 3:OF1–13. 10.1158/1541-7786.MCR-21-0924 35560224

[55] Yasinska IM Sakhnevych SS Pavlova L Selnø ATH Abeleira AMT Benlaouer O. The Tim-3-galectin-9 pathway and its regulatory mechanisms in human breast cancer. Front Immunol. (2019) 10:1594. 10.3389/fimmu.2019.01594 31354733 PMC6637653

[56] Milewska M Romano D Herrero A Guerriero ML Birtwistle M Quehenberger F. Mitogen-inducible gene-6 mediates feedback inhibition from mutated BRAF towards the epidermal growth factor receptor and thereby limits malignant transformation. PLoS ONE. (2015) 10:9859. 10.1371/journal.pone.0129859 26065894 PMC4466796

[57] Carén H Fransson S Ejeskär K Kogner P Martinsson T. Genetic and epigenetic changes in the common 1p36 deletion in neuroblastoma tumours. Br J Cancer. (2007) 97:1416–24. 10.1038/sj.bjc.6604032 17940511 PMC2360241

