# Supplementary material for: Association between night shift work and methylation of a subset of immune-related genes
Source: Front Public Health. 2023 Jan 12;10:1083826. doi: 10.3389/fpubh.2022.1083826 (PMC9877629; doi:10.3389/fpubh.2022.1083826)
Supplement: Supplementary file 1 [file Data_Sheet_1.pdf]

Supplementary Table 1. PCR cycling condition and primer sequences used for PCR amplification and pyrosequencing of target genes. Pyrosequencing assay information are reported according to UCSC Genome Browser, GRCh38/hg38 assembly.

Suppl. Table 1. Pyrosequencing assay information[1].

**Gene**	**Chromosome Position [1]**		**Primers (5′→3′)[2]: Forward (F) Reverse (R) Sequencing (S)**	**Sequence to Analyze (5′→3′)**	**T° Annea-ling**	**Ref**
	chr19:	F	Bio-GTGGATTTTTAAAGTTGTAAGTT			
ERVE	1442532	R	CCACCTAACAACCAAACTATAACA	A/GACCA/GACAACATCAACAAACTCA/GCA/GTCTTAAA	57°C	1
	-1442974	S	AAAAAAAAAATTTTTATTC			
	chr6:	F	Bio-AGGGGTAGGTTAGTAAGTAGGAGA			2
ERVFRD1	11111566	R	CCACCCCAAAAAAAAAAAATCC	CA/GCCTTACCTTCTTCA/GACTAAAAA	60°C	
	-11112154	S	CTAATAATAAAACACCTCTA			
	chr2:	F	Bio-AAAAGGAGGAAAAGTAAAGAAAGA			
ERVH	154867104 -	R	CCAAAAAAAAAAAATTTCACAAAA	AACA/GTATATATACAAA	50°C	3
	154867902	S	CAATTACTTCAAACCATCTA			
	chr3:	F	GTAAAGGGTTTGTGTTGAGGAG			
ERVK	101,691,853	R	Bio-ACTTATCCCACACCTCCAAC	TTTC/TGGTATAAAATT	52°C	3
	-101,692,992	S	TTTTGGGTAATGGAATG			
	chr12:	F	Bio-TGATTAATGGGATGTAGTTAAAGG			
ERVL	74063872	R	TCACCCATTAAAAAACACTCACA	CACA/GCTTCTTCCAAACCCCTAACCATCCAACA/GAAA	54°C	*
	-74066600	S	TATCACCAATTTTCCAAT			
	chr14:	F	TGTGGAGAAAGAAGTTTGATGTTA			
HERV-P	41599556 -	R	Bio-CCTTTTAATCTCTTCACTAATT	A/GTTTAAAAAAAAAACTAA ATTACTAT	52°C	3
	41600635	S	CCCTTTAAATCACAACC			
	chr7:	F	TTAGTTAGGAGGGGAGATTATAGG			
ERV-R	64999198	R	Bio-TCTCCCAATAATACCTTAAATCAC	TTC/TGGGTTGGGGGAGATTC/TGT	56°C	4
	-64999961	S	GTTGTGGGATTGGAGT			
	chr1:	F	TGTTATTGTTTATTTTTTRGGTTTA			
ERV9-1	1017305	R	Bio-TCTAAATTTTTTATTCCTCCC	TAATATTTATC/TGTTAA	52°C	5
	-1017518	S	TATATTGTTTTTATGAGTTG			
	chr7:	F	ATGGAGTTTAAGATGTAGTTTAAG			
ERVW-1	92097695	R	Bio-CAATCCCCCATCTCAACAA	C/TGTAGATTTTTGGATC/TGGTTTGTTAGTTTAC/TGATTT	55°C	6
	-92098707	S	AGTTTAAGATTAAGATTTAT			
	chr1:	F	Bio-GGAGGAAGAGGAGATGGGTT			
HRES1	229270597	R	CACTATCCCCAACCCTCTATCAC	TCA/GAAACTCAACCCCCAACA/GCCCCCACCTACCA/GCTCTA	57°C	7
	-229272748	S	CCCTCCCTCAAAAAAA			
	chr11:	F	TGGGGAGTTTTGTTAGGGGTTAG			
BIRC2	102217709	R	Bio-ACCCTCTCCACATTTCCTCTCTA	AAC/TGTAGC/TGGTTTAC/TGGTTTGTTAC/TGTTTTTTA	60°C	8
	-102217729	S	TAGGGTTTTTTAAAATTTA			
	chr20:	F	TGAAGAAGGGGGTGATGTTATAG			
FLRT3	14337486	R	Bio-TTCCAAAAAACTACCTACAAACCA	AC/TGTTAGAGGGGGC/TGAGAG	52°C	-
	-14337838	S	TAGATAGATGAGTTTTGTAA			
	chr17:	F	Bio-TTTTAGGTTTGGGTTGAGTATAGG			
GAL9	27630871	R	CCAAAACAACTTAACTATTATTCA	A/GACAACAAAAAAAAATAAATCC	52°C	-
	-27631218	S	TTATTCAATAAAAAAAAACC			
	chr8:	F	TGGTTTATTTTAGAGGTATTGTTT			
IDO1	39912969	R	Bio-TTCTCTTTTCTCCTTTTAATCATC	TC/TGTTTTTTTAGATGA	52°C	9
	-39913084	S	TAAAGAAGAAATTAAGTTGT			
	chr19:	F	Bio-TTTTGTGTAAGGGGTGTAGGAATA			
LPHN	13774983-	R	AAACTTTCAATTCCCTTCCCATAT	CA/GCAAATCTTACTTCTACA/GAAACCCCTTTT	56°C	-
	13775322	S	TATTAAAACCCTTTTTAAAC			
	chr1:	F	GGAGTTTGAATTTATGGGAAAT			
MIG6	8086334	R	Bio-ACAACCCTAAAAACCTCTCAAC	C/TGGC/TGTTGAGAGGTTTTTAGGGTTGTC/TGTAC/TGGTTT	56°C	10
	-8086790	S	GGGTTTTTGAGGAAAGTT			
	chr16:	F	TAGGGGAGAAGGGAATGGT			
NNLRC5	56988914	R	Bio-CTCTTTCCATCTCCCCCTTT	GC/TGTTC/TGTTTTTATTTAT	60°C	11
	-56989139	S	GGAGAAGGGAATGGTAGTA			
	chr10:	F	Bio-TAGTTTGAAAGAGAAGTTGAGAAAG			
SIRT1	69644144	R	AAAAATTTAAACCCCCATCA	CA/GTAACCCA/GCCCCATCA/GCCA/GCCCCCA/GCCCTC	55°C	-

	-69644392	S	AAATTTAAACCCCATCA			
XIAP	chrX: 123859829 -123860485	F<br>R<br>S	TTTTGTAGGGTAGGAGGTTGATTT<br>Bio-TCAAAACCTCAAAAAAACACC<br>GGGTAGGAGGTTGATTTT	TC/TGGTC/TGGGGGTGGGAGGGATAC/TGTC/TGGGGTTT	58°C	-

[1] As reported by the UCSC Genome Browser, GRCh38/hg38 assembly. The genomic cohordinates indicate the region where the assay was designed.

* Homo sapiens isolate HCL-4 endogenous virus HERV-L mRNA sequence chr12:74063872-7406660.

Supplementary Table 2. Association between NS (ever vs never; current vs former vs never) and NLRP3 and TNF alpha. Estimates were calculated by a multivariable regression model adjusted for age, BMI, smoking habits, oral contraceptive use, general health medications, and ELISA plate.

	Night shift work	Mean	95% CI		p-value
**NLRP3**	Ever	16,35	15,67	17,04	**0,0052**
	Never	14,73	13,84	15,62	
**TNF-alpha**	Ever	0,78	0,70	0,88	0,7947
	Never	0,76	0,65	0,89	

ever=64 never=28

	Night shift work	Mean	95% CI		p-value	Overall p-value	p-value for trend
**NLRP3**	Current	16,41	15,68	17,13	**0,0045**	**0,0209**	**0,0098**
	Former	16,18	15,10	17,25	**0,0469**		
	Never	14,74	13,86	15,62	**ref**		
**TNF-alpha**	Current	0,76	0,67	0,87	0,99	0,653	0,8988
	Former	0,84	0,69	1,02	0,7034		
	Never	0,75	0,64	0,89	ref		

current=44, former=20, never=28

Supplementary Table 3. Complete overview of the association between current NS work and methylation of all the genes of interest at selected BMI values.

Dependent variable: Gene-specific methylation	Interaction term	BMI cut-off for estimate of gene-specific methylation	Current NS work	Mean	SE	95% CI		current NS work (yes vs no) β=mean difference	SE	95% CI		p-value
**ERVE**	Current NS work * BMI P-value 0.0065	19.8 kg/m²	Yes	85.02	0.34	84.34	85.69	0.29	0.48	-1.23	0.66	0.5483
			No	84.73	0.29	84.15	85.31					
		22.02 kg/m²	Yes	84.60	0.27	84.07	85.12	-0.31	0.37	-0.42	1.05	0.3997
			No	84.91	0.24	84.44	85.38					
		**24.22 kg/m²**	**Yes**	**84.18**	**0.27**	**83.65**	**84.71**	**-0.91**	**0.38**	**0.17**	**1.66**	**0.0167**
			**No**	**85.09**	**0.26**	**84.58**	**85.61**					
		**29.14 kg/m²**	**Yes**	**83.25**	**0.51**	**82.25**	**84.26**	**-2.25**	**0.71**	**0.86**	**3.64**	**0.0016**
			**No**	**85.50**	**0.50**	**84.52**	**86.48**					
**ERVL**	Current NS work * BMI P-value 0.0286	**19.8 kg/m²**	**Yes**	**92.53**	**0.14**	**92.26**	**92.80**	**0.59**	**0.19**	**-0.98**	**-0.21**	**0.0027**
			**No**	**91.94**	**0.12**	**91.70**	**92.18**					
		**22.02 kg/m²**	**Yes**	**92.38**	**0.10**	**92.18**	**92.59**	**0.39**	**0.15**	**-0.69**	**-0.10**	**0.0099**
			**No**	**91.99**	**0.10**	**91.80**	**92.18**					
		24.22 kg/m²	Yes	92.23	0.11	92.02	92.45	0.20	0.15	-0.50	0.11	0.2080
			No	92.04	0.11	91.82	92.25					
		29.14 kg/m²	Yes	91.90	0.21	91.49	92.31	-0.25	0.29	-0.33	0.82	0.3972
			No	92.15	0.21	91.74	92.55					

**ERVW-1**	Current NS work * BMI<br>P-value 0.0329	19.8 kg/m²	Yes	94.41	0.20	94.01	94.80	0.36	0.28	-0.92	0.20	0.2072
			No	94.05	0.18	93.69	94.40					
		22.02 kg/m²	Yes	94.23	0.16	93.93	94.54	0.07	0.22	-0.51	0.36	0.7463
			No	94.16	0.14	93.88	94.45					
		24.22 kg/m²	Yes	94.07	0.16	93.74	94.39	-0.21	0.23	-0.24	0.67	0.3517
			No	94.28	0.16	93.97	94.59					
		**29.14 kg/m²**	**Yes**	**93.69**	**0.32**	**93.06**	**94.31**	**-0.85**	**0.44**	**0.00**	**1.71**	**0.0512**
			**No**	**94.54**	**0.30**	**93.94**	**95.14**					

References

1. Wu Z, Mei X, Zhao D, et al. DNA methylation modulates ERVE expression in CD4+ T cells from systemic lupus erythematosus patients. *J Dermatol Sci*. 2015;77(2):110-116. doi:10.1016/J.JDERMSCI.2014.12.004

2. Makaroun S, Himes K. Differential Methylation of Syncytin-1 and 2 Distinguishes Fetal Growth Restriction from Physiologic Small for Gestational Age. *AJP Rep*. 2018;8(1):e18-e24. doi:10.1055/S-0038-1627473

3. Dolci M, Favero C, Toumi W, et al. Human Endogenous Retroviruses Long Terminal Repeat Methylation, Transcription, and Protein Expression in Human Colon Cancer. *Front Oncol*. 2020;10:2145. doi:10.3389/FONC.2020.569015/BIBTEX

4. Lee JR, Ahn K, Kim YJ, Jung YD, Kim HS. Radiation-induced human endogenous retrovirus (HERV)-R env gene expression by epigenetic control. *Radiat Res*. 2012;178(5):379-384. doi:10.1667/RR2888.1

5. Vilahur N, Baccarelli AA, Bustamante M, et al. Storage conditions and stability of global DNA methylation in placental tissue. *Epigenomics*. 2013;5(3):341-348. doi:10.2217/EPI.13.29

6. Gimenez J, Montgiraud C, Oriol G, et al. Comparative methylation of ERVWE1/syncytin-1 and other human endogenous retrovirus LTRs in placenta tissues. *DNA Res*. 2009;16(4):195-211. doi:10.1093/DNARES/DSP011

7. Fali T, Le Dantec C, Thabet Y, et al. DNA methylation modulates HRES1/p28 expression in B cells from patients with Lupus. *Autoimmunity*. 2014;47(4):265-271. doi:10.3109/08916934.2013.826207

8. Tseng CC, Liao WT, Wong MC, et al. Cell lineage-specific methylome and genome alterations in gout. *Aging (Albany NY)*. 2021;13(3):3843. doi:10.18632/AGING.202353

9. Dewi DL, Mohapatra SR, Blanco Cabañes S, et al. Suppression of indoleamine-2,3-dioxygenase 1 expression by promoter hypermethylation in ER-positive breast cancer. *Oncoimmunology*. 2017;6(2). doi:10.1080/2162402X.2016.1274477

10. Lin CI, Du J, Shen WT, et al. Mitogen-inducible gene-6 is a multifunctional adaptor protein with tumor suppressor-like activity in papillary thyroid cancer. *J Clin Endocrinol Metab*. 2011;96(3). doi:10.1210/JC.2010-1800

11. Zeng Q, Chen X, Ning C, et al. Methylation of the genes ROD1, NLRC5, and HKR1 is associated with aging in Hainan centenarians. *BMC Med Genomics*. 2018;11(1). doi:10.1186/S12920-018-0334-1
